# Computer-Aided Detection of Retinopathy of Prematurity Severity in Preterm Infants *via* Measurement of Temporal Vessel Width and Angle

**DOI:** 10.3389/fped.2022.792724

**Published:** 2022-01-28

**Authors:** Yo-Ping Huang, Spandana Vadloori, Eugene Yu-Chuan Kang, Wei-Chi Wu

**Affiliations:** ^1^Department of Electrical Engineering, National Taipei University of Technology, Taipei, Taiwan; ^2^Department of Electrical Engineering, National Penghu University of Science and Technology, Penghu, Taiwan; ^3^Department of Information and Communication Engineering, Chaoyang University of Technology, Taichung, Taiwan; ^4^Department of Ophthalmology, Chang Gung Memorial Hospital, Taoyuan, Taiwan; ^5^Graduate Institute of Clinical Medical Sciences, College of Medicine, Chang Gung University, Taoyuan, Taiwan; ^6^College of Medicine, Chang Gung University, Taoyuan, Taiwan

**Keywords:** retinopathy of prematurity (ROP), vessel width, vessel angle, Radon transform, computer-aided diagnosis

## Abstract

Retinopathy of prematurity (ROP) is a retinal disorder that occurs in preterm infants with low birth weight and is the leading cause of preventable blindness in children. Early identification of high-risk patients and early diagnosis and timely treatment of ROP can substantially improve patients' visual outcomes. However, manual screening consumes both time and resources. Telescreening using retinal fundus images has the potential to reduce the burden engendered by the necessity of on-site screening. Recently, substantial progress has been made in using computer-aided diagnosis with retinal fundus images, and this approach has attracted considerable attention for the diagnosis of eye diseases. Abnormalities of and alterations in retinal blood vessels may relate to the occurrence and progression of ROP. In this study, we examined the hypothesis that ROP severity may be associated with the angle and width of arteries and veins. We computationally determined the artery–artery and vein–vein angles in the temporal quadrants—the temporal artery angle (TAA) and temporal vein angle (TVA)—under normal conditions and in different ROP stages. We also estimated retinal vessel width—temporal artery width (TAW) and temporal vein width (TVW)—by applying the Radon transform method to fundus images. Our results revealed significant decreases in TAA and TVA and increases in TAW and TVW with increasing ROP severity (all *P* < 0.0001).In addition, we observed positive TAA–TVA and TAW–TVW correlations (both *P* < 0.0001). The TAA was negatively correlated with the TAW (*r* = −0.162, *P* = 0.0314). These retinal vessel features may be useful in assisting ophthalmologists in the early detection of ROP and its progression.

## Introduction

Retinopathy of prematurity (ROP) is a vascular eye disease that occurs in preterm infants, especially those with low birth weight (<1,500 g) and young gestational age (<32 weeks). Detecting the disease in its early stage and conducting regular follow-ups are critical for preventing blindness. In the International Classification of Retinopathy of Prematurity (1, 2), ROP is categorized into five stages (stages 1–5) according to the severity of the disease: stage 1, involving a demarcation line at the juncture of the vascular and avascular peripheral retina; stage 2, involving a ridge; stage 3, involving neovascularization; stage 4, involving partial retinal detachment; and stage 5, involving total retinal detachment (2).

Screening for ROP to detect cases requiring treatment is crucial if a patient with ROP is to have a favorable outcome. Traditionally, ROP screening has been performed at the bedside through indirect ophthalmoscopy (3). Because of the limited personnel available for onsite screening, the use of tele-screening, in which a fundus image is captured using a wide-angle fundus camera, has increased (4). In one study, ROP features on fundus images could be identified with high accuracy (4).

Because of the recent development of artificial intelligence, automatic ROP diagnosis through the identification of plus disease has been achieved using deep learning technology (5–9). A few reports are available on retinal vessel angles and their relationship with ROP, especially as ROP progressively worsens (10, 11). Additionally, some studies have determined the width of retinal blood vessels. For example, a study determined the vessels' width by extracting the centerline after the segmentation of the vessels and using the pixel edges (12). In the other study, a Gaussian-based modeling approach has been employed to detect vessel width on the basis of centerline vessel intensity, and image processing techniques have been employed to determine vessel width on the basis of fundus images by using Gabor filters (13). However, these studies have applied image processing methods such as binarization and skeletonization and have used a manually set threshold for each image. The vessel segmentation and centerline extraction must be determined precisely in the middle of the vessel for accurate measurement of width, which is unlikely when automated image processing techniques are employed. The aforementioned methodologies are applicable only when the retinal images are of high quality with vessel contrast and full vessel development. However, in the case of fundus images of preterm infants, the vessels may not be completely developed, and the images are likely to be of low quality, which can result in inaccurate estimations of vessel width. Finally, the relationship between vessel angles and widths at various stages of disease severity has yet to be investigated.

To address these limitations, the present study used a Radon transform (RT)-based algorithm to determine the width of vessels automatically from fundus images of babies without ROP and babies with stage 1–3 ROP. The RT-based algorithm is robust and can reliably detect linear features even in the presence of noise (14, 15). Moreover, the algorithm can track thin vessel structures, for which the signal quality is low. The RT computation is executed through the integration of local intensities; accordingly, centerline smoothness is achieved using Gaussian smoothing based on spatial correlations. According to our review of the literature, the association between vessel angle and width has yet to be reported. In this study, we quantified the angles of the temporal artery and temporal vein and determined their correlations with the vessels' width as well as the extent to which they varied among preterm infants. We discovered the relationships of vessel angles and widths with an increase in ROP severity. Our study findings could offer information regarding the objective judgment of ROP severity and possibly complement the understanding of and improve screening strategies for ROP as well as aid its diagnosis and management.

## Materials and Methods

### Dataset Details

In this study, we used a dataset containing the fundus images of preterm infants who underwent eye screening for ROP at Chang Gung Memorial Hospital, Linkou, Taiwan. All images were captured by an ophthalmic technician using a RetCam imaging system (RetCam III, Natus, Pleasanton, CA, USA). The fundus exams were performed for infants with a body weight of ≤ 1,500 g or gestational age of ≤ 32 weeks and for selected infants with a body weight of 1,500–2,000 g or gestational age of 32 weeks with any unstable clinical condition; the ROP screening was performed in the fourth week after birth (16–18). Data from a total of 118 patients were used in this study. In brief, the data comprised 66 retinal images from 44 patients with no ROP, 28 images from 19 patients with stage 1 ROP, 33 images from 20 patients with stage 2 ROP, and 49 images from 35 patients with stage 3 ROP. All the data were captured between May 2013 and June 2019. Herein, the term “no ROP” refers to normal eye health without ROP. Details on the data are presented in Table 1.

**Table 1:** Details of data used in this study.

**ROP stage**	**No. of patients**	**No. of images**	**OS**	**OD**
No ROP	44	66	32	34
Stage 1	19	28	13	15
Stage 2	20	33	16	17
Stage 3	35	49	31	18

### Image Labeling

All fundus images were labeled by two senior expert ophthalmologists (EYK and WCW) in accordance with the guidelines of the International Classification of Retinopathy of Prematurity (1, 2). Furthermore, the vessels in the images were annotated as being an artery or vein at the main superior and inferior temporal branches in the retina.

### Image Analysis

The resolution of all fundus images was set as 1,600 × 1,200. The angles between the superior and inferior temporal arteries and between the superior and inferior temporal veins were measured using MATLAB software. The temporal artery angle (TAA) was determined by tracing a line along the superior and inferior temporal arteries. The vessel angle was calculated automatically at the point of intersection of these two straight lines by the algorithm using the inverse cosine function. A similar procedure was followed to measure the temporal vein angle (TVA) in each image. We calculated the TAA and TVA in all images in the dataset.

### Vessel Width Estimation

We used an RT-based algorithm to estimate the width of the vessels in all fundus images (15). The RT-based algorithm is robust and can detect linear features well even in the presence of noise. The intensity of an image generally fluctuates due to the presence of noise; however, when an RT-based algorithm is employed, these fluctuations are eliminated through an integration process. We used an RT-based linear feature detection algorithm to extract the centerline of vessels. The RT over a two-dimensional Euclidean space can be derived as follows:


(1) $$\begin{array}{r} R\left(\rho ,\theta \right)=\;\int_{-\alpha }^{+\alpha }\int_{-\alpha }^{+\alpha }g\left(x,\;y\right)\delta \left(\rho -x\cos\theta -y\;\sin\theta \right)dx\;dy \end{array}$$

where *g*(*x*,*y*) is the image intensity at position (*x*,*y*), δ is the Dirac delta function, ρ is the distance of the origin from the straight line along the line perpendicular to the straight line and going through the origin, and θ is the angle between the normal and the *x*-axis.

**Figure 1:**
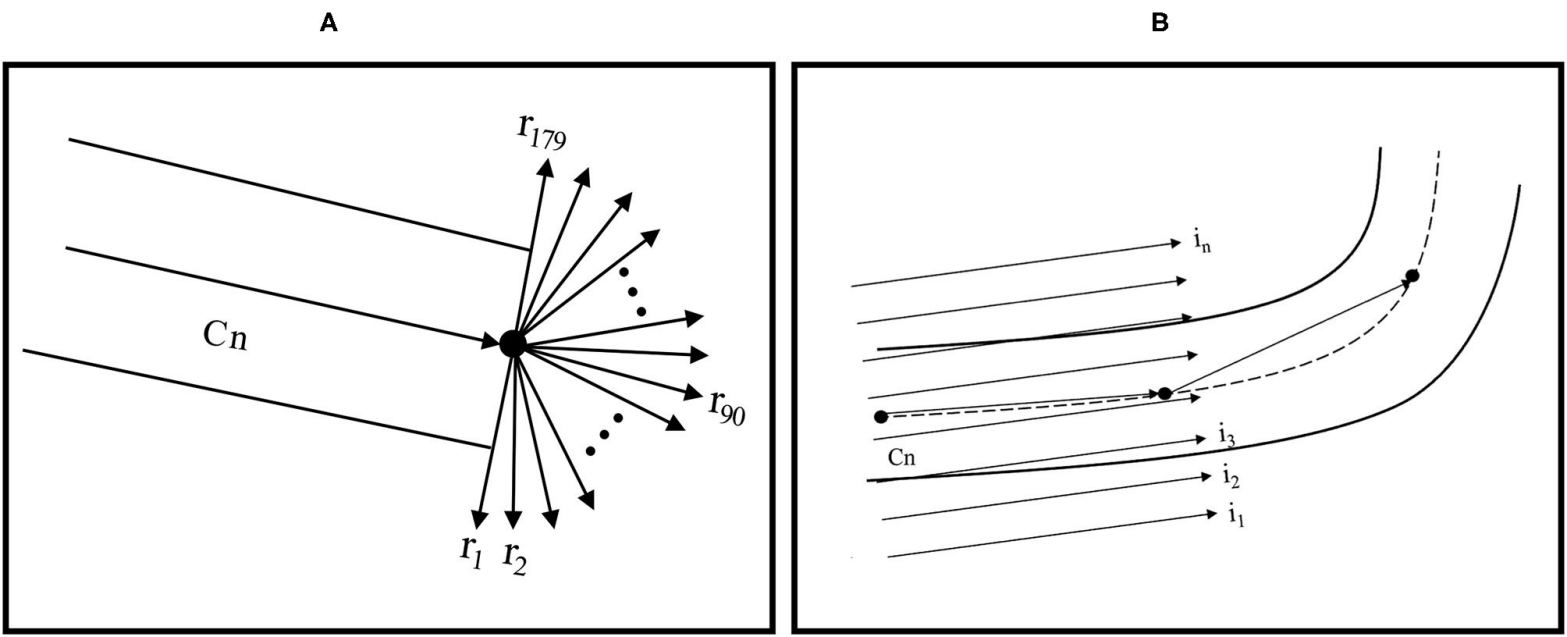
**(A)** RT feature extraction and centerline tracking and **(B)** computing RT features for vessel edge extraction with linear integrals.

The RT algorithm emphasizes the linear features in an image because the intensity is integrated along all possible lines in the image. The presence of the Dirac delta function forces the integration of *g*(*x*,*y*) along a line with the normal representation ρ = *x*cosθ + *y*sinθ (19). The parts of fundus images that show vessels can have more varied intensity than that in the background in the image and may contain areas of vessel curvature. Through the RT algorithm, vessel curvature and diameter could be estimated from previous values. Areas of curvature could be identified using a Gaussian process and by distinguishing positive from negative values by using means of zero. Initially, a center point on a vessel was selected manually and then defined as the centerline (Cn); the target direction point on the vessel was subsequently specified to predict the direction of Cn. The RT algorithm assigned a weight (ranging between 0 and 1) to each pixel on the basis of its distance from the selected Cn. The pixels nearer the selected Cn were assigned higher weights. A total of 179 vectors were formed near the selected Cn by computing the line integrals through cubic interpolation (Figure 1A). On the basis of the target direction and the similarity in intensity of the center pixels (compared with the vessel edge pixels), the corresponding Cn was calculated from the initial center point and moved a step forward. This process was continued until the target point was reached.

To determine vessel width, another RT with parallel line integrals was calculated in the vessel direction (Figure 1B). Because the boundaries of vessels are typically darker than the background of vessels, vessel edges could be determined. The diameter could be computed by calculating the Euclidean distance between the top-edge pixels and corresponding bottom-edge pixels. The average of these values was considered the vessel width.

We considered up to 100 pixels in length when determining vessel width because the TAA and TVA measurements were conducted near the optic disc. The superior and inferior artery and vein widths on the temporal side of the retina were quantified using the RT algorithm. We obtained the temporal artery width (TAW) and temporal vein width (TVW) by averaging the superior and inferior artery widths and vein widths, respectively. We obtained TAW and TVW values from all images, except for three images from patients with stage 2 ROP, from which we could not derive TAW values. Specifically, in these three images, we could accurately determine only the inferior artery width because an adjacent vein overlapped the artery in the images.

### Statistical Analysis

The data in the datasets were confirmed to be normally distributed before they were analyzed. The data were subjected to one-way analysis of variance (ANOVA) to identify significant differences among the no-ROP, stage 1, stage 2, and stage 3 groups. Subsequently, Tukey's multiple-comparison test was performed to compare subgroups. To determine TAA–TVA and TAW–TVW correlations, we employed Pearson's correlation, calculating the correlation coefficient *r*. Statistical analysis was conducted using GraphPad Prism software 9.0 (GraphPad Software, San Diego, CA, USA). *P* < 0.05 was considered statistically significant.

## Results

A total of 176 fundus images from 118 preterm infants were used in this study's analysis. All images were from different eyes; in addition, images from both eyes from 60 patients were included.

### Determination of Retinal Vessel Angle

**Figure 2:**
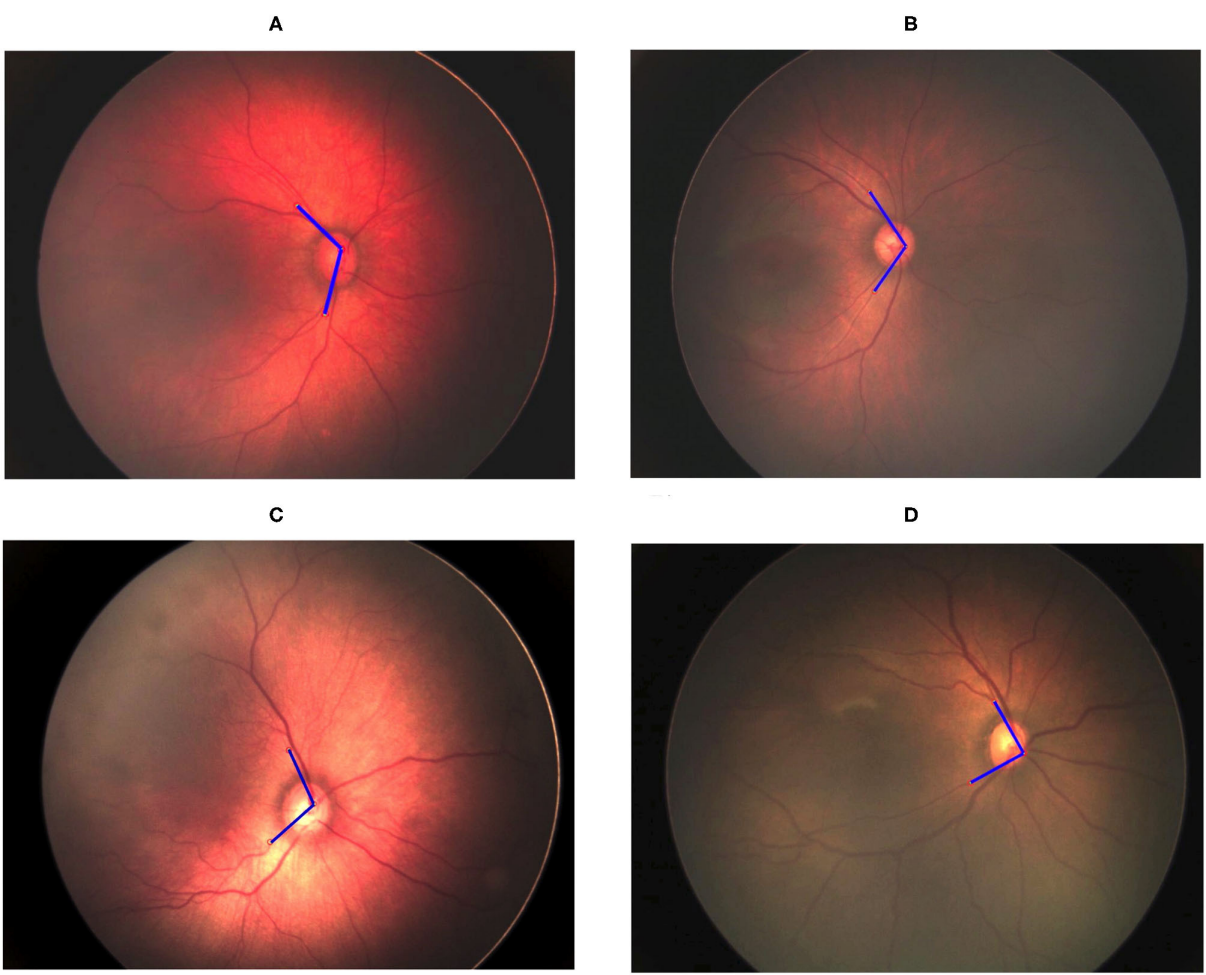
Gradual decrement in TAA from no ROP to stage 3 ROP. **(A)** No-ROP, **(B)** stage 1 ROP, **(C)** stage 2 ROP, and **(D)** stage 3 ROP groups, with angles 128.60°, 112.63°, 107.84°, and 89.50°, respectively. The blue lines indicate the direction of temporal retinal vessels.

**Figure 3:**
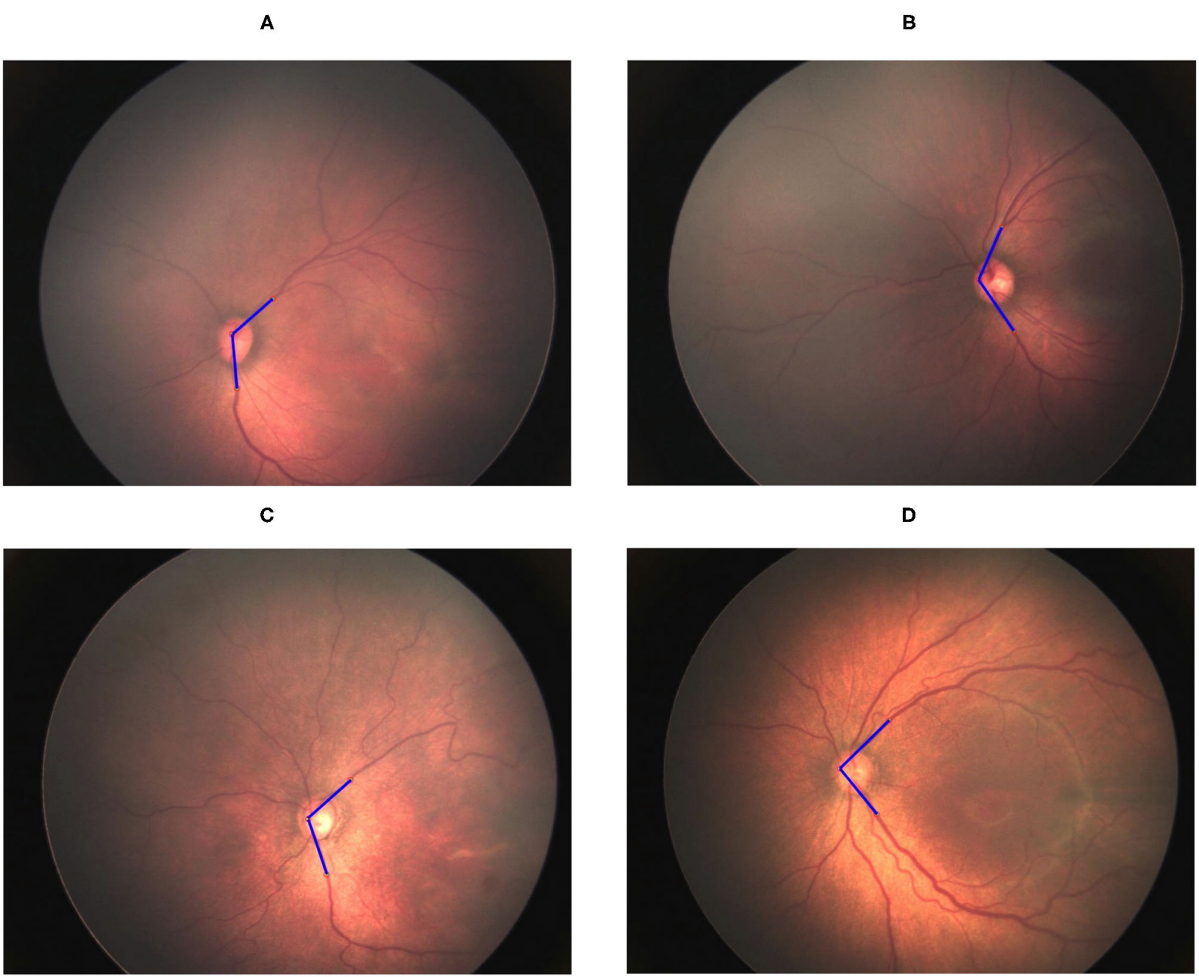
Gradual decrement in TVA from no ROP to stage 3 ROP. **(A)** No-ROP, **(B)** stage 1 ROP, **(C)** stage 2 ROP, and **(D)** stage 3 ROP groups, with angles 131.50°, 127.12°, 114.4°, and 95.18°, respectively. The blue lines indicate the direction of temporal retinal vessels.

The vessel angle was determined through vessel tracing. The mean TAA values in the no-ROP, stage 1 ROP, and stage 2 ROP groups were 122.42° ± 10.02°, 114.23° ± 14.95°, and 111.87° ± 10.52°, respectively. The mean TAA in the stage 3 ROP group was 92.72° ± 13.93°. The TAA was thus smaller at higher degrees of severity (no ROP vs. stage 3 ROP, *P* < 0.0001; Table 2). Similar observations were made for the TVA. The mean TVA values in the no-ROP, stage 1 ROP, stage 2 ROP, and stage 3 ROP groups were 123.96° ± 16.55°, 120.79° ± 18.65°, 115.26° ± 14.83°, and 102.86° ± 21.91°, respectively (*P* < 0.0001). Representative TAAs and TVAs in various ROP stages are displayed in Figures 2, 3, respectively.

**Table 2:** TAA and TVA in various stages of ROP.

		**TAA (degrees)**				**TVA (degrees)**			
**ROP stage**	**No. of images**	**Median**	**Min., Max**.	**Mean ±SD**	***P*-value**	**Median**	**Min., Max**.	**Mean ±SD**	***P*-value**
No ROP	66	122.44	98.53, 150.80	122.42 ± 10.02^bcd^	<0.0001^a^	123.75	88.62, 168.55	123.96 ± 16.55^d^	<0.0001^a^
Stage 1	28	117.12	87.48, 139.76	114.23 ± 14.95^be^		119.52	87.51, 157.29	120.79 ± 18.65^e^	
Stage 2	33	112.48	89.50, 135.30	111.87 ± 10.52^cf^		116.49	84.14, 147.49	115.26 ± 14.83	
Stage 3	49	92.15	57.66, 134.28	92.72 ± 13.93^def^		102.69	65.14, 160.09	102.86 ± 21.91^de^	

a *P-value was calculated using one-way ANOVA*. *bcdef: significant difference in the subgroup analysis between groups with the same letter*.

### Determination of Vessel Width

The TAW was obtained by averaging the widths of the superior and inferior arteries in all images, except for three images from a patient with stage 2 ROP. For those three images, we could only determine the inferior artery width because the superior artery was overlapped by an adjacent vein, which made it difficult to determine the width accurately. In these cases, we used only the inferior vessel width as the TAW in the analysis.

**Figure 4:**
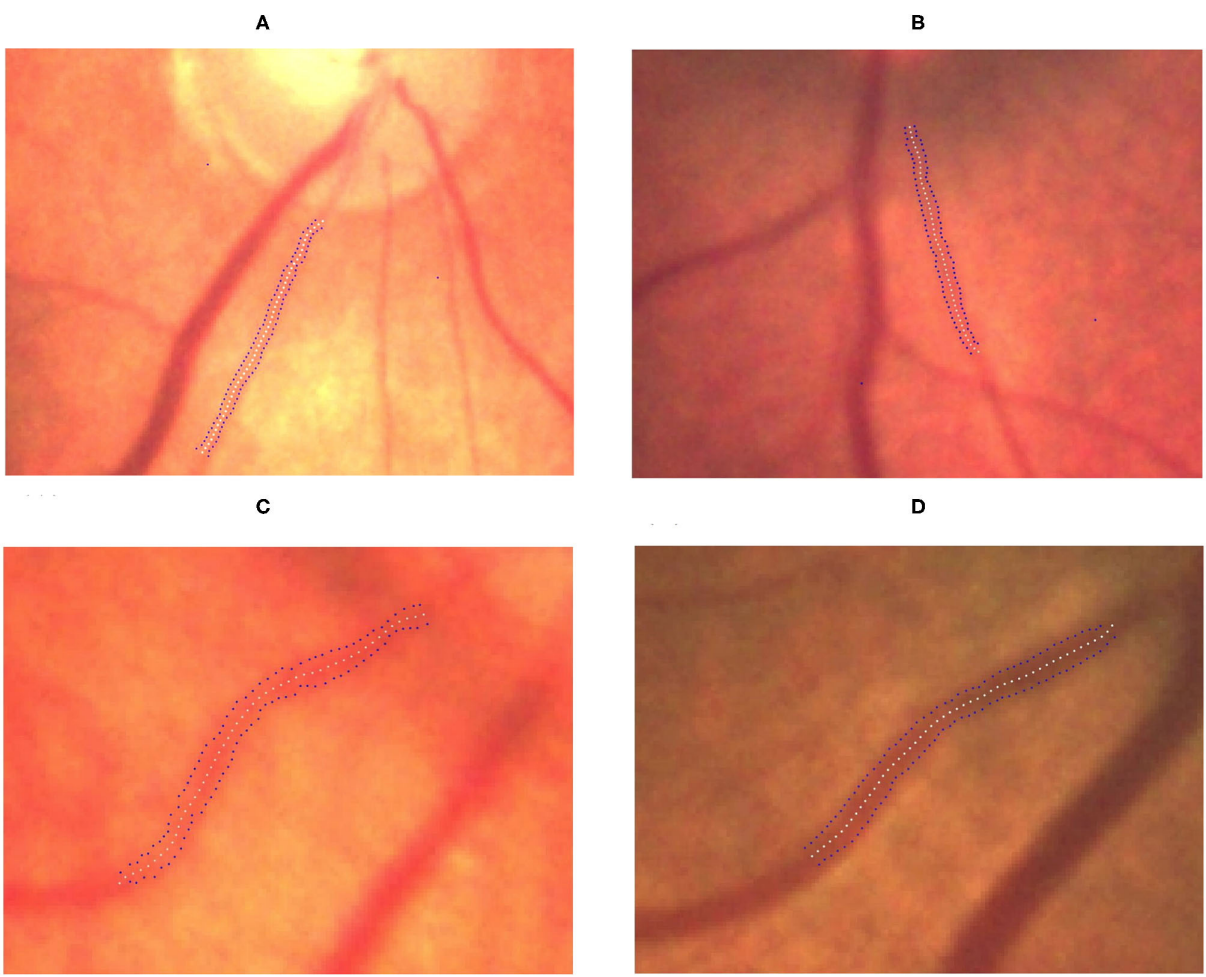
Gradual increment in TAW from no ROP to stage 3 ROP. **(A)** No-ROP, **(B)** stage 1 ROP, **(C)** stage 2 ROP, and **(D)** stage 3 ROP groups, with widths 4.15, 4.92, 5.14, and 6.28 pixels, respectively. The white dotted curve indicates the centerline of the vessel, and the blue dotted curves indicate the borderlines of the vessel.

**Figure 5:**
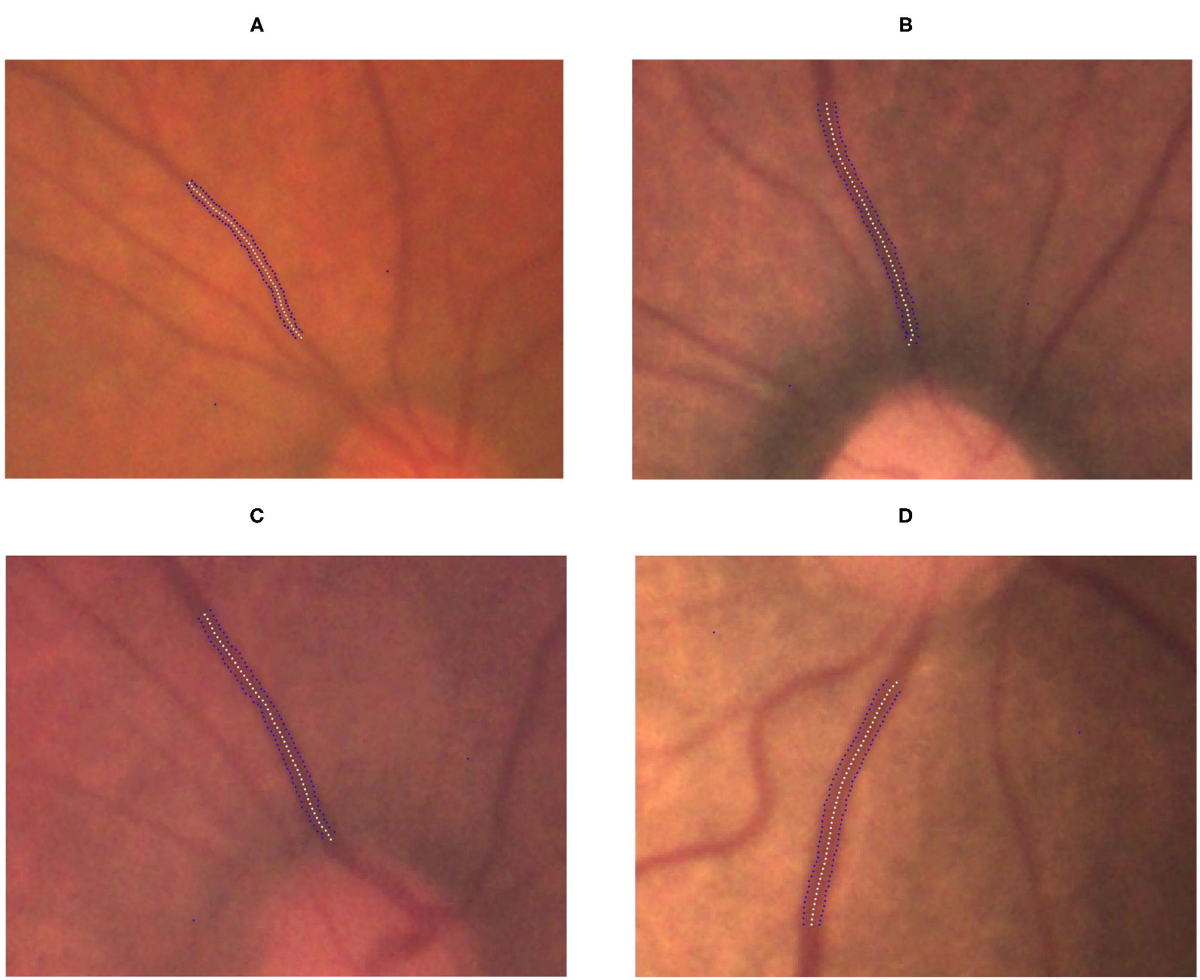
Gradual increment in TVW from no ROP to stage 3 ROP. **(A)** No-ROP, **(B)** stage 1 ROP, **(C)** stage 2 ROP, and **(D)** stage 3 ROP groups, with widths 5.14, 5.45, 5.76, and 6.99 pixels, respectively. The white dotted curve indicates the centerline of the vessel, and the blue dotted curves indicate the borderlines of the vessel.

We observed significant differences among the ROP severity groups in terms of both the TAW and TVW (*P* < 0.0001 and *P* = 0.0044, respectively; Table 3). In the subgroup analysis, the TAW and TVW in the stage 3 ROP group were significantly greater than those in the no-ROP group (both *P* < 0.05). Representative figures of the TAW and TVW are presented in Figures 4, 5, respectively.

**Table 3:** TAW and TVW in various stages of ROP.

**ROP stage**		**TAW (pixels)**				**TVW (pixels)**			
	**No. of images**	**Median**	**Min., Max**.	**Mean ±SD**	***P-*value**	**Median**	**Min., Max**.	**Mean ±SD**	***P-*value**
No ROP	66	4.31	3.03, 5.20	4.21 ± 0.51^b^	<0.0001^a^	5.66	4.26, 7.03	5.63 ± 0.60^b^	= 0.0044^a^
Stage 1	28	4.29	3.36, 5.18	4.22 ± 0.49^c^		5.75	4.78, 6.75	5.75 ± 0.52	
Stage 2	33	4.04	3.44, 5.07	4.10 ± 0.39^d^		5.59	4.04, 6.97	5.61 ± 0.68^d^	
Stage 3	49	4.59	3.46, 5.99	4.58 ± 0.59^bcd^		6.29	4.43, 6.98	6.03 ± 0.65^bd^	

a *P-value was calculated using one-way ANOVA*. *bcd: significant difference in the subgroup analysis between groups with the same letter*.

### Correlation of Vessel Angle With Vessel Width

**Figure 6:**
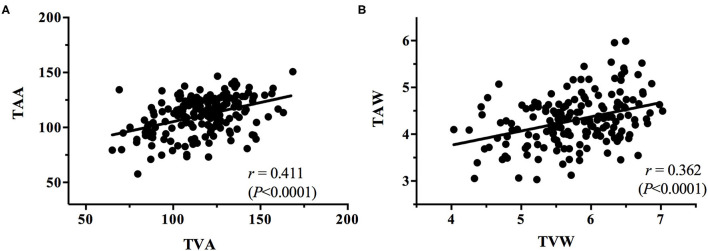
Plots of the correlations between **(A)** TAA and TVA and **(B)** TAW and TVW.

**Figure 7:**
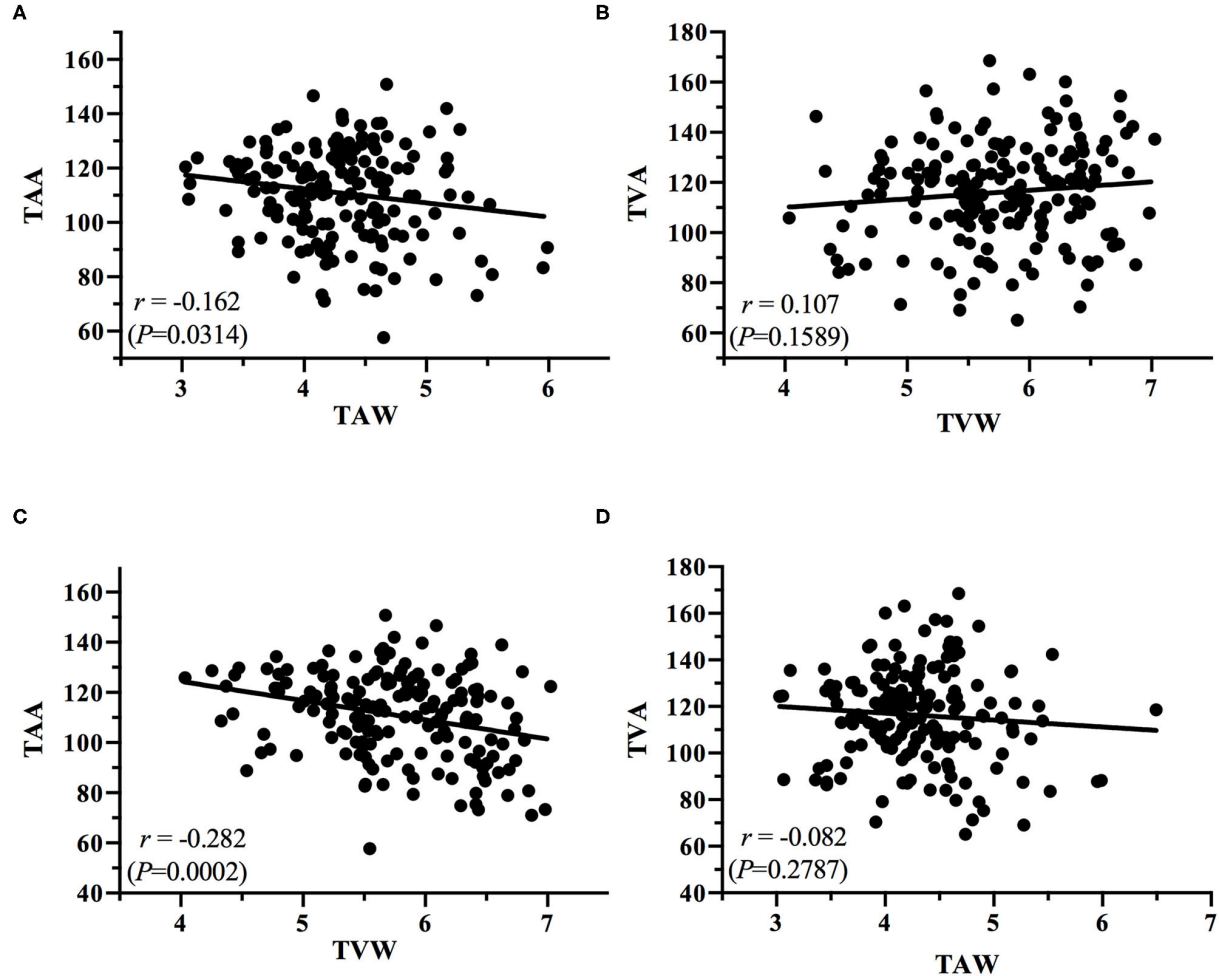
Plots of the correlations between **(A)** TAA and TAW, **(B)** TVA and TVW, **(C)** TAA and TVW, and **(D)** TVA and TAW.

The correlations between the vessel angles and vessel widths, for all stages of ROP, were plotted. Linear regression analysis of each correlation was conducted to determine the correlation coefficient *r* for the TAA–TVA, TAW–TVW, TAA–TAW, TVA–TVW, TAA–TVW, and TVA–TAW correlations irrespective of the stage (Figures 6, 7).

## Discussion

The present study obtained some notable findings. We discovered a significant decrease in the TAA and TVA and an increase in the TAW and TVW with increasing ROP severity (all *P* < 0.0001). In addition, we observed positive correlations between the TAA and TVA and between the TAW and TVW (*r* = 0.411, *P* < 0.0001; *r* = 0.362, *P* < 0.0001, respectively); we also noted negative correlations between the TAA and TAW (*r* = −0.162, *P* = 0.0314), the TAA and TVW (*r* = −0.282, *P* = 0.0002), and the TVA and TAW (*r* = −0.082, *P* = 0.2787). According to our review of the literature, this is the first study to investigate the relationship between temporal vessel angles and widths for various stages of ROP. Subtle retinal vessel changes are crucial because they can assist clinicians in the early detection of ROP and its progression. Serial information on vessel angle and width can indicate whether ROP is progressing or regressing. The identification of ROP progression to a higher stage in an eye gives surgeons time to react so that treatment can be performed without delay once stage treatment-requiring ROP is reached. In the future, the identified features could be incorporated into an algorithm for automatic ROP diagnosis in the era of telemedicine and artificial intelligence.

Previous studies have attempted to investigate how vessel angles vary in preterm infants with and without ROP (10, 11). In one of these studies, the TVA was manually measured by drawing an axis perpendicular to the fovea and optic disc center by using points on the vessels that vertically divided the fovea (10). The study focused on measuring retinal vein angles only. In another study, the optic disc center was used to measure the artery and vein angles semiautomatically (11); the results revealed no significant differences between the no-ROP and mild-ROP (stages 1 and 2) groups, but they indicated a significant difference in vessel angle between the no-ROP and severe-ROP (stage 3) groups. In the present study, we initially applied the strategy employed in that study to our dataset, using the optic disc center to find the vessel angles. However, we observed that the strategy did not always correctly reflect the vascular angle in images because not all the vessel intersection points were located at the optic nerve center. Hence, in this study, we modified the methodology for measuring the angles. The superior and inferior vessels were traced back toward the optic disc to their point of intersection. The angle at this intersection point was considered the vessel angle. We noted that the current method is more reliable and precise in accurately determining the vessel angle.

The present study revealed an inverse relationship between ROP stage and the temporal vascular angle. The mean TAA and TVA decreased gradually from no ROP to stage 1, stage 2, and stage 3 ROP. These results indicate the progressive stretching of retinal vessels through fibrovascular proliferation as ROP progressed. Wilson et al. (10) reported that the mean differences in TVA between no-ROP and stage 3 ROP groups were 6.1° and 0.9° in the right and left eyes, respectively. Wong et al. (11) indicated that the differences in median TAA and TVA between no-ROP and stage 3 ROP groups were 16° and 13°, respectively. In the present study, the differences in median TAA and TVA between the no-ROP and stage 3 ROP groups were 30.29° and 21.06°, respectively (Table 2), considerably greater than the previously reported differences (10, 11). Wong et al. (11) hypothesized that after premature birth, the arteriole angle is more strongly affected than the venule angle. In our study, the retinal artery angle and retinal vein angle were equally significantly affected by ROP disease progression, and these angles were correlated with the vessel width.

In addition to a decrease in vessel angle, we observed variations in vessel width for the various stages of ROP. Retinal vessel dilatation, which is part of the parameters of plus disease, may be related to an increased vascular endothelial growth factor level in ROP eyes (20, 21). We estimated the vessel widths in all stages of ROP by using an RT-based algorithm that can detect linear features precisely even in the presence of noise (14, 15). Our results reveal significantly larger TAW and TVW values in the stage 3 ROP group than those in the no-ROP group (Table 3). However, the difference between the no-ROP and stage 1 ROP groups and that between the no-ROP and stage 2 ROP groups were not significant. Our results suggest that in the early stages (stages 1 and 2) of ROP, vessel width may not be significantly affected by vessel angle alterations but that when the disease has reached stage 3, the width of the vessels becomes significantly altered.

The angles and widths of the arteries and veins were correlated. The vessel angle decrease (TAA vs. TAW; TVA vs. TVW) was associated with an increase in vessel width from the stage of no ROP to stage 3 ROP. Positive TAA–TVA and TAW–TVW correlations were identified (Figure 6), whereas negative TAA–TAW (Figure 7A), TAA–TVW (Figure 7C), and TVA–TAW (Figure 7D) correlations were observed. These data show that only one of these measurements must be used to obtain a meaningful outcome in future studies.

The key findings of this study are as follows. First, we confirmed the hypothesis that ROP severity is associated with the angle and width of arteries and veins. The relationship between the temporal vessel angles and widths is a novel finding and, to our knowledge, has not been reported by previous studies. Second, other studies have measured the retinal angle centered in the optic disc, which is inaccurate because not all vessels intersect at the disc center. We measured the vessel angle as the vessels exited the disc margin and extended the line until intersection; this method can better reflect the actual angles of the vessels. Third, the measurement of the temporal vessel width was performed using RT, a new approach that provided reliable outcomes. The subtle retinal vascular changes that were discovered as ROP progresses can give clinicians objective feedback and aid clinical decision-making.

This study has some limitations. First, the dataset used contained fewer images from patients with stage 1 and 2 ROP compared with the number for no ROP and stage 3 ROP, which may have caused the lack of a difference in retinal vessel features between the no-ROP, stage 1 ROP, and stage 2 ROP groups. Second, artery and vein annotations had to be performed manually because of the large variation in the branching pattern of the retinal vessels, and this manual annotation is a time-consuming process. Third, because we did not sedate the infants while taking photographs, the infant may have moved during image capture, leading to the quality of the images not always being high quality and to difficulty in accurately determine vessel width.

In conclusion, this study demonstrated that the severity of ROP is related to retinal vessel angles and widths. We discovered an inverse relationship between ROP severity and retinal vessel angle, but we found a positive correlation between ROP severity and retinal vessel width. These data are valuable and could serve as indicators of disease progression or regression. Further study in this area is required. With the further development of artificial intelligence technology, the subtle changes discovered in the present study could be integrated with scientific computer-aided approaches to give clinicians an objective judgment of the progression or regression of ROP.

## Data Availability Statement

The raw data supporting the conclusions of this article will be made available by the authors, without undue reservation.

## Ethics Statement

The studies involving human participants were reviewed and approved by Chang Gung Memorial Hospital. Written informed consent to participate in this study was provided by the participants' legal guardian/next of kin.

## Author Contributions

Y-PH, SV, E-YK, and W-CW: conceptualization, investigation, and writing—review and editing. Y-PH and SV: methodology, formal analysis, and writing—original draft preparation. SV: software. Y-PH, E-YK, and W-CW: validation and supervision. Y-PH and W-CW: resources, project administration, and funding acquisition. E-YK and W-CW: data curation and visualization. All authors have read and agreed to the published version of the manuscript.

## Funding

This study was funded in part by the Ministry of Science and Technology, Taiwan, under grant MOST108-2221-E-027-111-MY3, in part by a joint project between the National Taipei University of Technology and the Chang Gung Memorial Hospital under Grant NTUT-CGMH-110-01, by joint projects between the Chang Gung Memorial Hospital and National Taipei University of Technology Joint Research Program (CGMH-NTUT-2020-No. 01 and CGMH-NTUT-2021-No. 1) This study was also supported by Chang Gung Memorial Hospital research grants (CORPG3L0111 and CMRPG3L0151) and Ministry of Science and Technology, Taiwan, research grants (MOST 109-2314-B-182A-019-MY3).

## Conflict of Interest

The authors declare that the research was conducted in the absence of any commercial or financial relationships that could be construed as a potential conflict of interest.

## Publisher's Note

All claims expressed in this article are solely those of the authors and do not necessarily represent those of their affiliated organizations, or those of the publisher, the editors and the reviewers. Any product that may be evaluated in this article, or claim that may be made by its manufacturer, is not guaranteed or endorsed by the publisher.
